# Comprehensive Annotation of Bidirectional Promoters Identifies Co-Regulation among Breast and Ovarian Cancer Genes

**DOI:** 10.1371/journal.pcbi.0030072

**Published:** 2007-04-20

**Authors:** Mary Q Yang, Laura M Koehly, Laura L Elnitski

**Affiliations:** 1 Genome Technology Branch, National Human Genome Research Institute, National Institutes of Health, Rockville, Maryland, United States of America; 2 Social and Behavioral Research Branch, National Human Genome Research Institute, National Institutes of Health, Rockville, Maryland, United States of America; University of California San Diego, United States of America

## Abstract

A “bidirectional gene pair” comprises two adjacent genes whose transcription start sites are neighboring and directed away from each other. The intervening regulatory region is called a “bidirectional promoter.” These promoters are often associated with genes that function in DNA repair, with the potential to participate in the development of cancer. No connection between these gene pairs and cancer has been previously investigated. Using the database of spliced-expressed sequence tags (ESTs), we identified the most complete collection of human transcripts under the control of bidirectional promoters. A rigorous screen of the spliced EST data identified new bidirectional promoters, many of which functioned as alternative promoters or regulated novel transcripts. Additionally, we show a highly significant enrichment of bidirectional promoters in genes implicated in somatic cancer, including a substantial number of genes implicated in breast and ovarian cancers. The repeated use of this promoter structure in the human genome suggests it could regulate co-expression patterns among groups of genes. Using microarray expression data from 79 human tissues, we verify regulatory networks among genes controlled by bidirectional promoters. Subsets of these promoters contain similar combinations of transcription factor binding sites, including evolutionarily conserved ETS factor binding sites in *ERBB2, FANCD2,* and *BRCA2.* Interpreting the regulation of genes involved in co-expression networks, especially those involved in cancer, will be an important step toward defining molecular events that may contribute to disease.

## Author Summary

Promoters are regulatory regions that control transcription of genes. A special class of promoters, known as *bidirectional* promoters, regulates expression of two genes instead of one. These promoters are situated between two adjacent genes whose transcription start sites are physically within 1,000 bp and oriented in opposite directions. Bidirectional promoters are found repeatedly in the genome, suggesting an important biological significance for this regulatory configuration. We developed an algorithm to map bidirectional promoters using data from a comprehensive list of transcribed sequences known as expressed sequence tags, or ESTs. This approach improved the number of previously characterized bidirectional promoters by 300%. Included in the new data are bidirectional promoters that regulate expression of genes implicated in somatic cancers. For instance, ten well-recognized genes implicated in breast and ovarian cancers were identified as having bidirectional promoters. Three of the genes are further related by having duplicate copies of the same binding site for a transcription factor within their bidirectional promoters. These binding sites are conserved among species, providing greater evidence that they are functionally important. This example, in which similar regulatory structures are used to control genes involved in cancer, illustrates how data can be mined from the comprehensive set of bidirectional promoters. Within this manuscript, we show statistical evidence that many cancer genes are regulated by bidirectional promoters. These promoters will be a valuable dataset for studying the role of gene regulation in tumor development.

## Introduction

Bidirectional gene pairs are defined as two genes arranged head-to-head (adjacent 5′ ends) on opposite strands of DNA and within 1,000 bp of one another [1]. The definition of 1,000 bp is supported by analyses of Trinklein et al. [2], which show an enrichment of genes whose 5′ ends are on opposite strands and within 1,000 bp and no enrichment of genes with 5′ ends on the same strand at this distance. The sequences between the transcription start sites of bidirectional gene pairs are known as bidirectional promoters, and they influence expression of both adjacent genes. Bidirectional promoters represent a regulatory construction that is utilized repeatedly in the human genome, with 1,352 known examples [2].

Despite substantial interest in these promoters [1–3], the biological significance of this regulatory architecture is not well-established. The bidirectional arrangement is conserved among species, suggesting that it is functionally important [2]. Furthermore, a strand-specific pattern of the nucleotides in these promoters [3] could play a role in binding regulatory proteins. For instance, SP1-binding sites have been suggested as key regulators of CpG island promoters [4]. If groups of bidirectional promoters are similarly regulated, then distantly located genes under the control of these promoters should show common expression patterns. Additional regulatory factors must also be involved, because genes that form a bidirectional pair are not always expressed together. Experimental studies show that within a pair, genes can be expressed in a mutually exclusive manner [2]. How the intervening promoter sequence preferentially activates one gene versus another is currently unknown.

The categories of genes regulated by these promoters imply that specialized transcription factor binding sites could regulate expression of these genes. For instance, bidirectional promoters regulate DNA repair genes [1]. DNA repair genes frequently play a role in cancer that is elicited through mutations in their coding sequences. However, another plausible scenario toward the development of cancer comes from the misregulation of bidirectional promoters, especially if they contain common transcription factor binding sites. These sites would be candidates to explain a general mechanism in which regulatory disruptions at bidirectional promoters could play a pivotal role in cancer.

We developed a new algorithm to comprehensively map bidirectional promoters in the human genome using spliced expressed sequence tags (ESTs). The extended set of bidirectional promoters elucidates relationships among genes regulated by bidirectional promoters through co-expression networks. We show evidence that a subset of these promoters, which regulate genes implicated in breast and ovarian cancers, contain transcription factor binding sites in common.

## Results

### Mapping and Validation of Bidirectional Promoters

Although ESTs frequently represent truncated forms of full-length mRNAs, large EST collections can provide robust evidence of uncharacterized bidirectional promoters through a combination of features. For instance, the UCSC (University of California Santa Cruz) Human Genome Browser (http://www.genome.ucsc.edu) annotates the direction of transcription for each EST. Also, EST data contains thousands of transcripts captured by oligo-capping techniques, providing enrichment in the 5′ sequences of genes [5]. Given this information, we hypothesized that neighboring, spliced ESTs, which are transcribed in opposite directions and initiate within 1 kb of one another, are unlikely to represent disconnected pieces of the same transcript, and thereby represent either a bidirectional or a *cis*-antisense overlapping gene pair.

**Figure 1:**
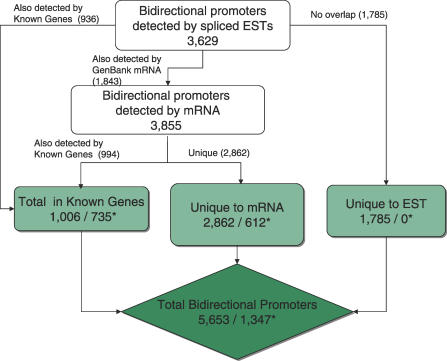
Identification of Bidirectional Promoters The diagram depicts how bidirectional promoters were identified from the UCSC Human Genome Browser annotations, including Known Genes, mRNA, and spliced ESTs. The number of previously identified bidirectional promoters for each category is marked with an asterisk (*) for comparison. Promoters identified from more than one source were counted only once. Priority was given to promoters identified from Known Genes followed by GenBank mRNA, and then spliced ESTs.

Our initial screen to identify bidirectional promoters partitioned the output according to its presence within annotation tracks of the UCSC Genome Browser. Because the spliced ESTs are the most complicated dataset, priority was given to promoters identified in more highly curated datasets, such as Known Genes and GenBank mRNA, over the spliced human ESTs [6]. Our dataset was compared with the previously published collection of bidirectional promoters [2], confirming 99.6% of that collection and contributing an additional 300% (Figure 1). The majority of newly identified bidirectional promoters came from the GenBank mRNA track (2,862) and from the spliced EST data (1,785). Overall, the spliced ESTs and mRNA identified nearly equivalent amounts of these promoters (3,529 or 3,855, respectively), but priority was given to the mRNA annotations for direct comparison to the previous dataset.

Overall, we found strong evidence for 5,653 bidirectional promoters in the human genome. All promoters defined only by EST data were scrutinized by comparison with GenBank mRNA and UCSC Known Genes to detect complementary support for the direction of transcription. In this way, we determined that 1,100 of the EST transcripts flanking a bidirectional promoter are novel transcripts, overlapping nothing in either reference track. Another 974 overlap an mRNA or Known Gene, and extend the 5′ position of that reference gene by more than 100 bp. Some of these extension events add a full 5′ UTR to the reference gene and cover extreme distances to join an upstream alternative promoter. For example, the bidirectional promoters identified upstream of *ERBB2* (*HER2/NEU*) or novel gene *AK094318* lie 10 kb and 140 kb away, respectively, from additional downstream promoters (unpublished data).

To validate the EST gene pairs using biological evidence, all intervening regions were examined for two features known to associate with promoters: TAF250 binding (or TAF1 [7]) and CpG islands. Greater than 50% of the bidirectional promoters identified solely from the spliced EST data have TAF250 present at both transcription start sites (and 70% have at least 1), and more than 90% have CpG islands. Thus, the bidirectional promoters identified using the spliced EST data can represent biologically active promoters. Furthermore, to verify biological activity of these predicted bidirectional promoters, each was compared with existing 5′ capped transcription data (known as CAGE) [8]. This independent experimental data confirmed the expression of pairs of flanking transcripts for 91%, 58%, and 65% of the promoters in the Known Genes, mRNA, and EST datasets, respectively (Figure S1).

### Significant Overrepresentation of Bidirectional Promoters Associated with Cancer-Related Genes

The strong enrichment of bidirectional promoters associated with DNA repair genes [1] implies a possible link between this regulatory mechanism and genes participating in cancer pathways. Therefore, we examined DNA maintenance, metabolism, and repair genes from the COSMIC database (Catalogue of Somatic Mutations in Cancer; [9]) for an association with bidirectional promoters. Of 302 genes implicated in somatic cancer, 45% had bidirectional promoters. The association is statistically significant with a *p*-value of ≤10e-6. In comparison, when sampled randomly from the genome, only 31% of human genes were associated with a bidirectional promoter. Furthermore, without our EST and mRNA analyses, only 12% of the genes on the COSMIC list could be identified as having a bidirectional promoter.

Other lists of somatic cancer genes were examined, including breast and colon cancer genes [10]. A significant enrichment of bidirectional promoters was found for somatic breast cancer genes (with a *p*-value of 0.01), but not somatic colon cancer genes. These data confirm an association between bidirectional promoters and somatic cancer genes, and indicate that the type of cancer is relevant.

We further examined bidirectional promoters associated with genes implicated in uterine and ovarian cancers. For instance, 16 genes have been implicated in Type I and II endometrial (uterine) cancers ([11] and references therein), and we found that eight of them have bidirectional promoters (Table S1). Although a comprehensive list of all genes implicated in somatic ovarian cancer is not available, a brief scan identified ten genes associated with mutations or mis-regulation in ovarian (and breast) cancer that have bidirectional promoters (*BRCA1, FANCA, BARD1, FANCF, TP53, BRCA2, CHEK2, ERBB2, FANCB,* and *FANCD2;* Table 1). This is the first report of bidirectional promoters for all of these genes except *BRCA1* [12] and for these cancer-related genes as a group. Bidirectional promoters for several of the genes (for example, *BRCA2*) were identified solely from the spliced EST data. Although the partner to *BRCA2* is not a well-recognized gene, it was originally identified through rigorous screening by RT-PCR and sequence conservation to validate its presence in the cell [13].

**Table 1:**
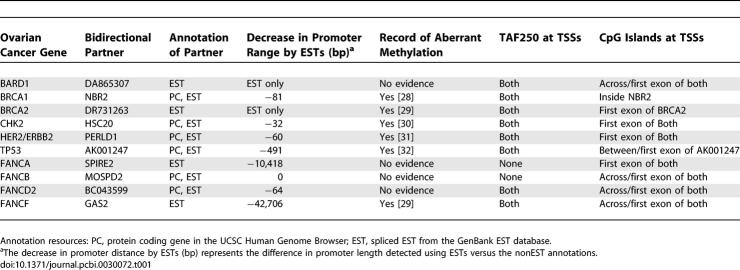
Somatic Ovarian Cancer Genes Regulated by Bidirectional Promoters

### Relationships among Genes Regulated by Bidirectional Promoters

**Figure 2:**
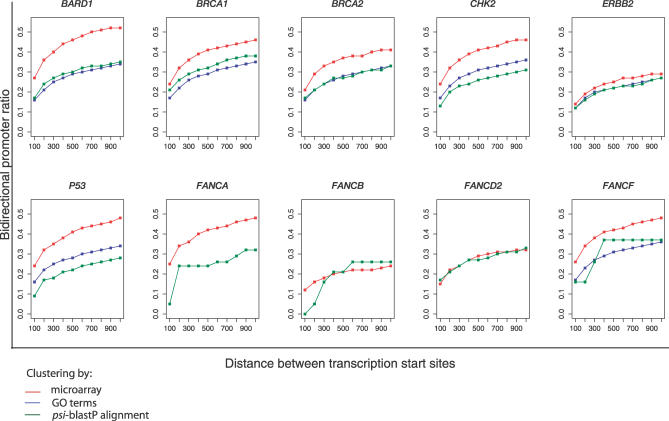
Clustering Analysis To Assess Bidirectional Promoters Clustering methods include microarray expression data (red), GO categories (blue), and *psi*-blastP alignments (green). The plots show the ratio of bidirectional promoters calculated for the cumulative number of genes found at each promoter distance. The named breast or ovarian cancer gene served as the reference gene for each type of cluster. The computational approach used to count the number of bidirectional promoters within each cluster is described in Methods. GO annotations (blue) were omitted when no data was available.

Many of the somatic ovarian cancer genes function in DNA surveillance and repair. Although none of these ten genes flank the same bidirectional promoter, several of them interact in multisubunit complexes implicated in breast or ovarian cancer, suggesting a large network of genes regulated to achieve coordinated functions. To address if bidirectional promoters are limited to a particular protein function, structural domain, or expression pattern, the ovarian cancer genes were clustered with other genes in the genome. Clustering was based on: (1) functional similarity—i.e., using a classification system of the biological function of all protein-coding genes called gene ontology (GO [14]); (2) amino-acid sequence and secondary-structure similarity by comparative alignment through *psi*-blastP [15]; or (3) similar expression patterns based on microarray data collected from 79 human tissues [16]. Each type of cluster was assessed for the ratio of bidirectional promoters. These data were graphed on the same *x*-axis, representing the distance between the transcription start sites for the observed bidirectional promoters. In this way, all data in the plot displayed the characteristic asymptotic curve demonstrated in Trinklein et al. [2], illustrating that the majority of these start sites were separated by ∼300 bp. Furthermore, the ratio of bidirectional promoters was calculated for the cumulative number of genes at each distance. Eight of the ten ovarian cancer genes had a higher ratio of bidirectional promoters in the co-expression clusters than in the *psi*-blastP clusters or GO clusters (Figure 2). This result indicated that the set of genes regulated by bidirectional promoters extended beyond a particular function or structure. Therefore, a larger analysis of co-expression patterns could provide meaningful information about co-regulated groups of bidirectional promoters.

### Expression Networks Enriched in Bidirectional Promoters

**Figure 3:**
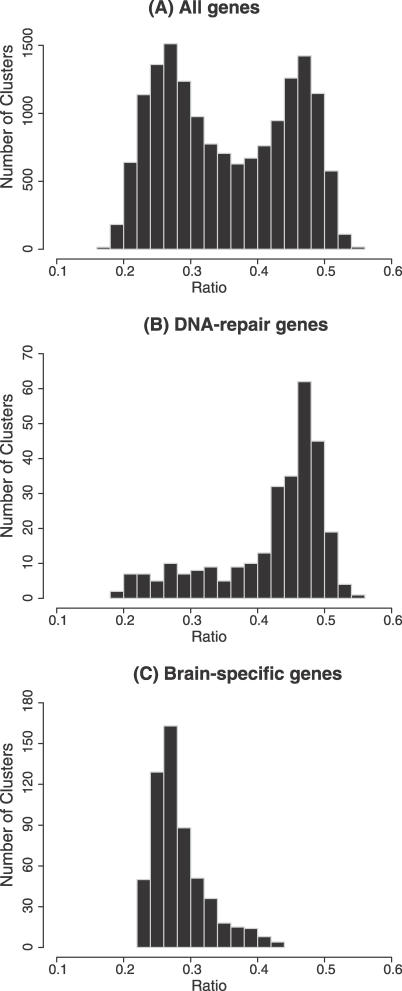
Frequency of Bidirectional Promoters Each of 16,078 genes represented in the microarray data was grouped with its 500 most-related genes into an expression cohort. The ratio of bidirectional promoters in each expression cohort was plotted for three categories: (A) all genes, (B) DNA-repair genes, or (C) brain-specific genes.

To examine coordinated regulation of bidirectional promoters more thoroughly, we clustered all 16,078 protein-coding genes into co-expression groups using the 79 tissues in the Novartis GNF human tissue arrays [16]. Each reference gene was clustered with its closest 500 expression neighbors. By analogy, we confirmed that the human beta-globin gene is most closely co-regulated with the human alpha-globin gene, whose finely balanced expression levels are necessary for physiological homeostasis. Each expression cluster was scored for the ratio of its 500 genes associated with a bidirectional promoter. These data were plotted as the number of clusters versus the ratios, and showed two peaks—containing high or low ratios of bidirectional promoters relative to the genome average (Figure 3). The ratios ranged from 0.16 to 0.56. Extreme enrichment occurred for clusters containing mRNA processing proteins and mitochondrial proteins (with a *p*-value of ≤1e-100 for clustering of these categories compared with random genome sampling; see Table S2). Extreme depletion of bidirectional promoters occurred in co-expression clusters containing sensory perception genes (*p*-value ≤1e-12 from random clustering; see Table S2). Thus, there is strong evidence of coordinated expression among sets of genes regulated by bidirectional promoters. This analysis revealed an additional ovarian cancer gene with a strong enrichment of bidirectional promoters in its co-expression cluster, *OVCA2* (“candidate tumor suppressor in ovarian cancer 2”), a serine hydrolase, which had a ratio of 0.56. In a smaller comparison, clusters containing DNA repair genes were skewed toward enrichment for bidirectional promoters, whereas brain-specific gene clusters were skewed towards depletion (Figure 3B and 3C).

**Figure 4:**
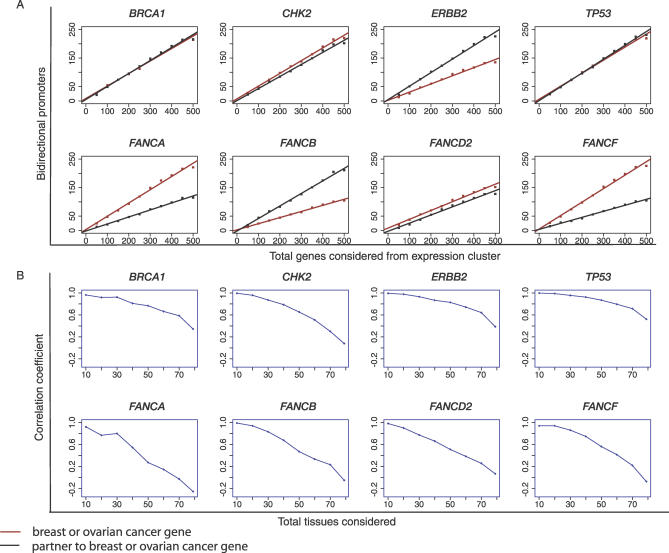
Co-Regulation of Bidirectional Promoters (A) The number of bidirectional promoters is graphed for the breast and ovarian cancer genes (solid brown line) and their bidirectional partner genes (solid black line). All graphs have the same axes: (*x*) the ranking of genes in the expression cluster according to their distance from the target gene and (*y*) the number of bidirectional promoters identified at this distance. (B) The correlation coefficient between each breast and ovarian cancer gene and its partner was calculated using the expression data in all 79 tissues. The correlation coefficients are plotted from the largest to smallest values; therefore, the tissue identity may change between plots to accommodate the ordering scheme.

Results of the expression-clustering analysis suggested that bidirectional promoters should not be considered under an umbrella classification for one large regulatory network, nor should they be divided into thousands of gene pairs. As illustrated by the ten ovarian cancer genes, the relationships are intermediate between the two extremes. These genes showed that one or both members of the pair had significant clustering with other genes regulated by bidirectional promoters. In two cases, a cancer-related gene clustered less well than its partner. Despite this clustering, the bidirectional promoters were able to regulate each flanking gene separately, as shown by the decreasing correlation coefficient for expression between the pairs of genes (Figure 4A and 4B). These data demonstrated that seven of the ten ovarian cancer genes ranked in the top quartile of all 16,078 genes for clustering with other genes regulated by bidirectional promoters (*p*-value for enrichment over the genome average ≤0.05; Table S3). Additionally, the enrichment was significant compared with a control group of monodirectional promoters in those same clusters [i.e., promoters that regulate genes arranged in a head-to-tail fashion that fell within 1,000 bp of their neighbor (Figure S2)]. For further comparison, bidirectional promoters were under-represented in expression clusters of brain-specific genes, with an average ratio of 0.23 (Figure S2).

The relationships between the ten ovarian cancer genes were mapped using multidimensional scaling (MDS) to model a co-expression network based on the overlap of genes in the clusters.

Looking within the clusters, we saw seven of the ovarian cancer genes were co-expressed with the same set of six genes, and additional genes were shared among smaller numbers of ovarian cancer genes to suggest an interconnected expression network. The probability of finding the same six genes in seven of the 16,078 clusters was statistically unlikely if their expression patterns were completely unrelated (less than 1/10,000; see Methods). Two of the shared genes, *MLH1* and *ITGB3BP,* have bidirectional promoters; they are known to function in ovarian or breast cancer, respectively [17–18]. The bipartite graph (showing relationships to and from the reference gene) resulting from the MDS analysis illustrated the similarity in co-expression patterns as the distances between the reference genes (Figure S3). The ten cancer genes separated into three groups based on similarity of their co-expression clusters. The groups contained *BRCA1*, *FANCA,* and *BARD1*; *FANCF*, *TP53,* and *BRCA2*; and *ERBB2, FANCB,* and *FANCD2*. The groupings suggested that subsets of genes could have transcription factor binding sites in common.

### Shared Transcription Factor Binding Sites

**Figure 5:**
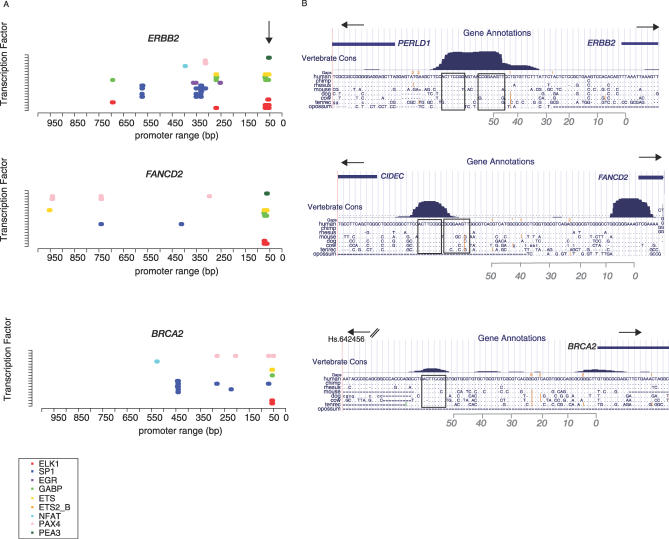
Conserved ETS Factor Binding Sites (A) Transcription factor-binding sites are shown for the bidirectional promoters of *ERBB2, FANCD2,* and *BRCA2*. The vertical arrow points to the binding site preferred by the ETS factor family member ELK1. The legend indicates the identity of all binding sites. (B) The sequence level view of these ETS factor binding sites are shown as snapshots from the UCSC Human Genome Browser. Horizontal arrows indicate the direction of transcription. Position 0 in (A) corresponds to the transcription start site of the cancer genes in (B). The annotation track labeled “Vertebrate Cons” represents the multiple-sequence alignment and conservation track for the eight-way vertebrate genome comparison at the UCSC Human Genome Browser. The putative ELK1-recognition sequences are boxed. The partner to *BRCA2* is not within the range shown.

Relationships between these cancer-related genes were further supported by the presence of shared transcription factor binding sites. We mapped 741 consensus motifs [19] to find the most frequent occurrences in these bidirectional promoters. The most common motifs were Sp1, NFAT, EGR-1, PAX4 (or RXR), and the ETS factor family member, ELK1. Compared with the set of brain-specific genes, the ten cancer genes had a 10-fold larger representation of consensus ETS factor binding sites. These sites comprised ELK1-binding sites, plus overlapping recognition sequences for other ETS factor family members (PEA3 and GABP). Confirming the hypothesis of a regulatory connection between some of these genes, tandem conserved binding sites for ELK1 mapped to the same position in *ERBB2* and *FANCD2*, 50 bp upstream of the transcription start site (Figure 5). A single conserved site containing this same DNA sequence was found at nearly the same distance upstream of *BRCA2*. *BRCA1* also had tandem sequences for the core of ETS factor binding sites (GGAA) near this position (unpublished data). Additionally, ETS factor binding sites were present as a trio with SP1 and PAX4/RXR binding sites in eight of nine ovarian cancer gene promoters. The brain-specific genes had no proximal ETS factor binding sites and fewer occurrences of the trio of binding sites (22% versus 77% in the ovarian cancer genes).

Additional promoter sets supported the importance of ETS factor binding sites. For example, *MLH1* and *ITG3BP,* which were co-expressed with seven of the ovarian cancer genes, had two and three consensus ETS factor binding sites within 1 kb of their transcription start sites, respectively. Returning to the 16,078 co-expression clusters containing 500 genes each, three clusters with the highest ratio of bidirectional promoters showed a distinct enrichment of the consensus ELK1 motif compared with three co-expression clusters with the lowest proportion of bidirectional promoters (the comparison returned a *p*-value <0.001 in a Chi-square test). The ELK1 motif was present in an average of 26% of bidirectional promoters in the enriched dataset and only 6% of bidirectional promoters in the depleted set. Thus, ELK1 binding sites were not universally present in all bidirectional promoters.

## Discussion

The spliced EST data significantly increases the number of bidirectional promoters that can be identified in the human genome and reveals uncharacterized 5′ UTRs associated with some protein-coding genes. These 5′ ends may have been previously overlooked due to a systematic under-representation of the 5′ ends of genes in the curated datasets. The identification of these 5′ termini of genes further enables a precise determination of the position of the associated promoter. Although the number of genes associated with a bidirectional promoter seems large, many genes produce transcripts by selecting from multiple promoters [20]. Given the extreme diversity in the choice of 5′ ends for many transcripts, bidirectional promoters may support only a minority of transcripts from a single locus. Furthermore, the large number of bidirectional promoters reported here represents a portion of an unknown total number of transcription start sites in the genome.

Relationships among the ten ovarian cancer genes are supported by their co-expression profiles and shared transcription factor-binding sites. For instance, all ten genes and several co-expressed genes contain ETS-family binding sites in their promoters. The role of ETS proteins is central to cancer biology [21]. Although numerous ETS proteins can bind the same core motif, specialized regulatory instructions may be dictated through different family members. Thus, computational approaches cannot conclusively implicate any particular ETS protein family member. Nevertheless, the patterns of binding motifs are reminiscent of the collection of binding sites recognized by the TNF-alpha enhanceosome, where combinations of binding sites (ETS, NFAT, SP1, and c-Jun) recruit CBP/p300 and RNA Pol II. Occupancy at these binding sites varies in response to the environmental cue and through competition for overlapping binding motifs [22].

A precise list of all genes involved in somatic mutations in cancer is difficult to define. Nevertheless, we were able to show enrichment of bidirectional promoters in cancer-related gene sets. The presence of shared binding sites provides a basis for explaining coordinated expression among these promoters. The evolutionary conservation of the binding sites argues that they are under selection to fulfill a specific functional role. The identical placement of these sites upstream of the promoters implies the mechanism of action is similar at all three promoters.

### Potential for Mis-Regulation of Bidirectional Promoters

In addition to common expression patterns, bidirectional promoters have the propensity for mis-regulation. Five of the nine breast and ovarian cancer genes have evidence of aberrant methylation affecting their expression levels in somatic tumors (see Table 1). Moreover, methylation of the *MLH1* bidirectional promoter [23] is almost exclusively responsible for cases of sporadic mismatch-repair deficiency in colon cancer [24]. This gene is located at the center of our shared expression network. The development of sporadic cancer is consistent with the loss of important functions of genes regulated by bidirectional promoters (e.g., genome stability, transcription, cell cycle control, nucleotide binding, tumor suppression, or mitochondrial stress response). These functions extend beyond the role of DNA repair, and are consistent with a much broader category of functions involved in the response to DNA damage. Loss of any of these cellular activities could lead to a neoplastic phenotype. Additionally, these functions argue that mis-regulation of bidirectional promoters could extend beyond breast and ovarian malignancies.

Epigenetic modifications associated with sporadic breast and ovarian cancers—such as silencing of bidirectional promoters—suggest a parallel mechanism by which regulatory disturbances could affect similar sets of genes as do mutations. Methylation could impede the binding of a protein (such as an ETS factor family member), putatively affecting the expression of two genes regulated by the bidirectional promoter. It has been previously shown that methylation of a bidirectional promoter inhibits expression of both associated genes [25]. We propose that bidirectional promoters be explored comprehensively as targets of aberrant methylation. We have identified a number of genes implicated in sporadic breast and ovarian cancers that are regulated in this manner.

## Materials and Methods

### EST mapping.

The strand information of spliced ESTs was obtained from the table “hg17.estOrientInfo” at the UCSC Human Genome Browser (http://www.genome.ucsc.edu), where the direction of transcription for each EST in the genome is determined based on consensus intronic splice junctions. Coordinates for both spliced ESTs, Known Genes, and GenBank mRNA were downloaded from the UCSC Human Genome Browser. Prior to obtaining the final dataset of bidirectional promoters, our curation process removed questionable EST pairs (Figure 1), resulting in 5,653 promoter regions identified in this analysis. Curation was based on the following steps. Initial classifications were made for the intergenic or intragenic location of the promoter region. Each promoter was subsequently placed into confidence levels on the basis of supporting ESTs. A binary decision tree was used for sorting. The tree forked into left and right branches, representing the intragenic or intergenic promoters, respectively. Parallel classification schemes were implemented along both branches of the tree to assign confidence levels to the predictions. For instance, ESTs were required to display majority agreement for the orientation of transcription in the region. Additional considerations were used to resolve overlapping *cis*-transcription units caused by densely packed overlapping genes or embedded transcripts. Overlapping but antisense transcription units required comparison with the protein coding gene annotations for further validation. The predictions were accepted only if there was an absence of conflict with the protein coding gene annotations. Successive rounds of annotation produced the final list of gene pairs. Seven hundred eight bidirectional promoters fell within introns of protein coding genes to identify alternative promoters that direct transcription of both a shorter form of a protein coding gene and a divergent gene that is antisense to it. These were examined on a case-by-case basis to ensure their legitimacy. Additional pairs of transcripts were identified in which one EST and one UCSC Known Gene are present on each side, accounting for ∼159 gene pairs. In total, the new analysis identified nearly four times as many bidirectional gene pairs as were previously published (5,653 versus 1,352).

### Expression clustering.

GNF Gene Expression Atlas 2 data was downloaded from the UCSC Human Gene Sorter ([26]; http://www.genome.ucsc.edu) in gene clusters of the most 500 similarly expressed genes with a reference gene. The relationships were calculated by UCSC as a weighted sum of differences in log expression ratios. The similarity of each gene's expression profile to all others was extracted from the table “hg17.gnfAtlas2Distance”. For each of the 16,078 genes listed, we obtained the nearest 500 genes, with a threshold score based on the weighted sum of differences of less than 1.0. The ratio of bidirectional promoters in each group of 500 was determined by comparison to our reference list of bidirectional promoters determined using the EST binary classification scheme described in the section *EST mapping* and the bidirectional genes identified in the UCSC Known genes and GenBank mRNA annotation tracks. For each cluster of 500 genes, we converted the proportion of bidirectional promoters in the list into a slope (Figure S2 or Figure 4) for visualization. The ratio of bidirectional promoters was compared with monodirectional promoters in the ovarian cancer gene clusters and brain specific gene clusters (as in Figure S2), and for pairs of the ovarian cancer genes that flank the same bidirectional promoter (as in Figure 4). Bidirectional and monodirectional promoters had a limit of 1,000 bp from their neighbors and were picked from the same expression cohorts.

To assess the probability of finding the same six genes in seven co-expression clusters, we simulated the situation 10 million times. In the simulations, the maximum number of genes in common in at least six of the seven sets was 1, and this occurred in 0.01% of the samples (i.e., 1 in 10,000).

### Multidimensional scaling.

The bidirectional promoters that are associated with the breast and ovarian cancer genes were considered an affiliation network and were transformed into a bipartite graph. Geodesic distances between genes were computed, and the geodesic distance matrix was scaled using the metric MDS algorithm in UCINET 6. The distance between the ovarian cancer genes in Figure S3 represents their similarity based on the number of shared genes found in the other ten cancer gene clusters.

### TAF250 binding and CpG island mapping.

For Taf250 and CpG islands assessment, we downloaded tables from the UCSC Human Genome Browser with the chromosome coordinate to indicate the position of the Taf250 binding site (hg17.LI Ng val TAF1) and CpG islands (hg17.cpgIslandExt). These tables contained data for validated Taf250 binding sites in IMR90 cells. The validation used a condensed array of sites that were previously observed to be positive in a whole-genome scan. Coordinates from each dataset were compared with the bidirectional promoters to find those that overlap.

### Statistical methods.

A Chi-square test of independence verified the association of bidirectional promoters and somatic breast cancer genes with a *p*-value of 0.01. The same test was used to assess the association of bidirectional promoters with DNA repair genes and is described in the main text.

In Figure 3, we observed two intersected, approximate normal distributions representing the frequency of bidirectional promoters clustered in the microarray expression profile for 16,078 genes. The intersection of these two distributions is at ∼0.32. Applying Pearson's Chi-square test to the 302 DNA repair genes and 546 brain specific genes, χ2 = 511, df = 1, *p* < 2.2e-16.

Functional classification of genes clustered into coordinately regulated groups was accomplished using the GOStat server [27]. The software calculates a *p*-value representing the probability that the counts could have appeared randomly using a Chi-square or Fisher's Exact test depending on the sample size.

The correlation coefficient for the expression of paired genes was calculated after sorting the tissues according to the similarity of the expression levels across all 79 tissues. The correlation coefficient was calculated for subsets of the datapoints, beginning with the first ten tissues and incrementally adding ten tissues each time.

## Supporting Information

Figure S1 Verification of Bidirectional Promoters Using RIKEN Transcript Data The bar graphs show the number of bidirectional promoters that were confirmed using the independently generated CAGE dataset from RIKEN. The data are separated into Known Genes, mRNA, and spliced ESTs for consistency with Figure 1. Blue bars represent CAGE transcripts for both sides of the bidirectional promoter. Green bars show the number of promoters with CAGE evidence for only one flanking transcript. Red bars indicate bidirectional promoters that are not detectable using the CAGE data. The number of promoters with support on both sides is printed above the blue bars.
(317 KB PDF)

Figure S2 Bidirectional Promoters versus Monodirectional Promoters Co-expression clusters were examined for the number of genes regulated by bidirectional or monodirectional promoters. Bidirectional promoters are graphed in red and monodirectional promoters in green. (A) depicts the ten breast and ovarian cancer genes. (B) shows ten brain-specific genes. All transcription start sites were within 1 kb of each other. This data was collected from the UCSC Known Genes only.
(153 KB PDF)

Figure S3 Relationships between Co-Expression Clusters Produced from MDS Each node on the graph represents one of the ten breast and ovarian cancer genes or an aggregate group of genes shared among clusters. The groups had to contain at least four genes to be included in the graph. The distance between points is the geodesic distance, illustrating overall similarity in expression clusters by placing more related cancer genes closer together. The outside nodes are associated with a smaller number of shared genes, whereas the central nodes are associated with a larger number of them. Numbers were used to label nodes representing groups of shared genes, whereas names were used to label the cancer reference genes. The profiles containing the *MLH1* and *ITGB3BP* genes lie in the center of the figure.
(329 KB PDF)

Table S1 Endometrial Cancer Genes with Bidirectional Promoters (28 KB DOC)

Table S2 Expression Clusters Over- and Under-Represented in Bidirectional Promoters (69 KB DOC)

Table S3 Bidirectional Promoters in Breast and Ovarian Cancer Gene Co-Expression Clusters (30 KB DOC)

## Abbreviations

EST: expressed sequence tag
MDS: multidimensional scaling
UCSC: University of California Santa Cruz
