# Supplementary material for: Comprehensive Annotation of Bidirectional Promoters Identifies Co-Regulation among Breast and Ovarian Cancer Genes
Source: PLoS Comput Biol. 2007 Apr 20;3(4):e72. doi: 10.1371/journal.pcbi.0030072 (PMC1853124; doi:10.1371/journal.pcbi.0030072)
Supplement: Supplementary file 1 [file pcbi.0030072.sg001.pdf]

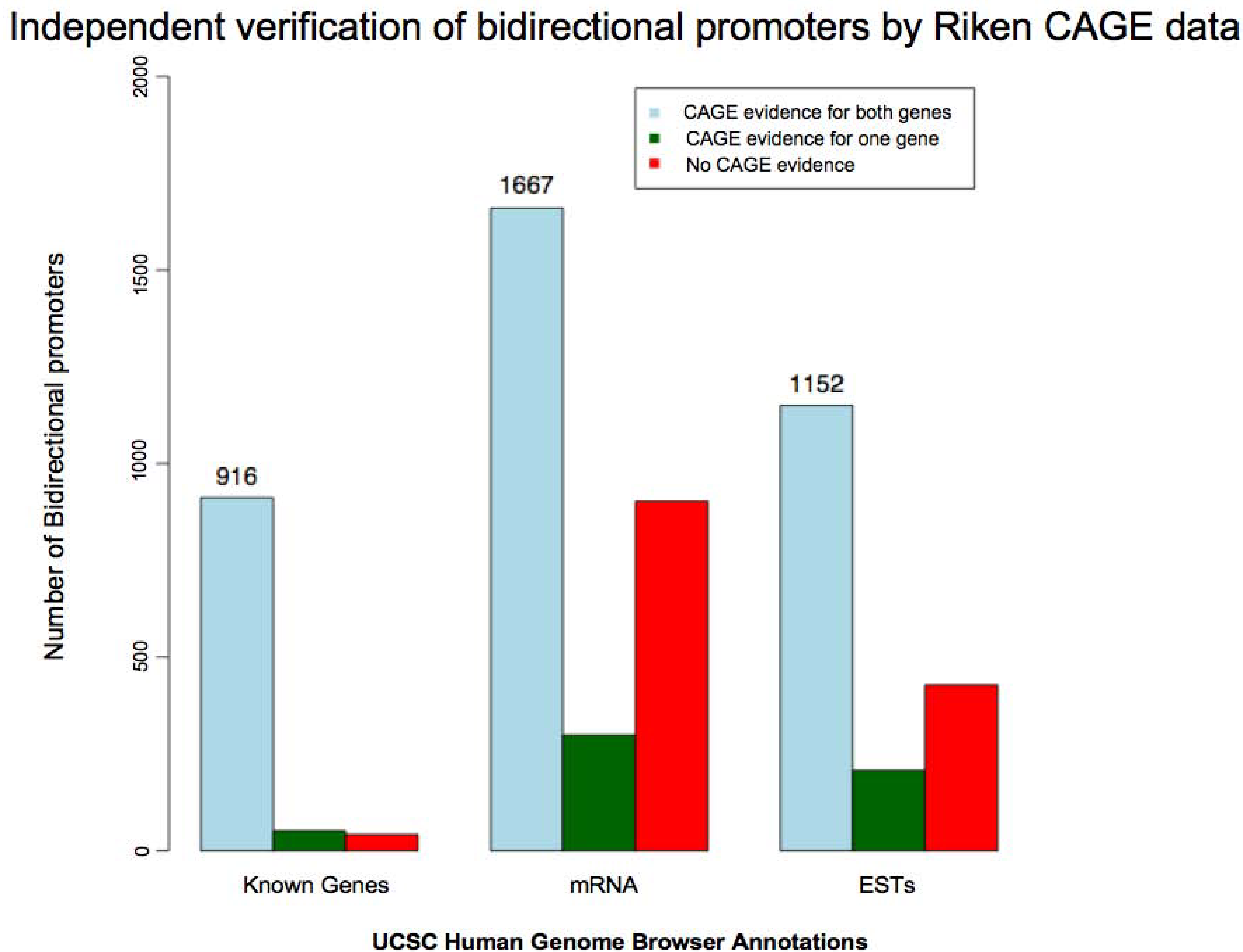
Independent verification of bidirectional promoters by Riken CAGE data
Number of Bidirectional promoters
0
500
1000
1500
2000
CAGE evidence for both genes
CAGE evidence for one gene
No CAGE evidence
916
1667
1152
Known Genes
mRNA
ESTs
UCSC Human Genome Browser Annotations

Figure S1
