# Supplementary material for: Comprehensive Annotation of Bidirectional Promoters Identifies Co-Regulation among Breast and Ovarian Cancer Genes
Source: PLoS Comput Biol. 2007 Apr 20;3(4):e72. doi: 10.1371/journal.pcbi.0030072 (PMC1853124; doi:10.1371/journal.pcbi.0030072)
Supplement: Supplementary file 2 [file pcbi.0030072.sg002.pdf]

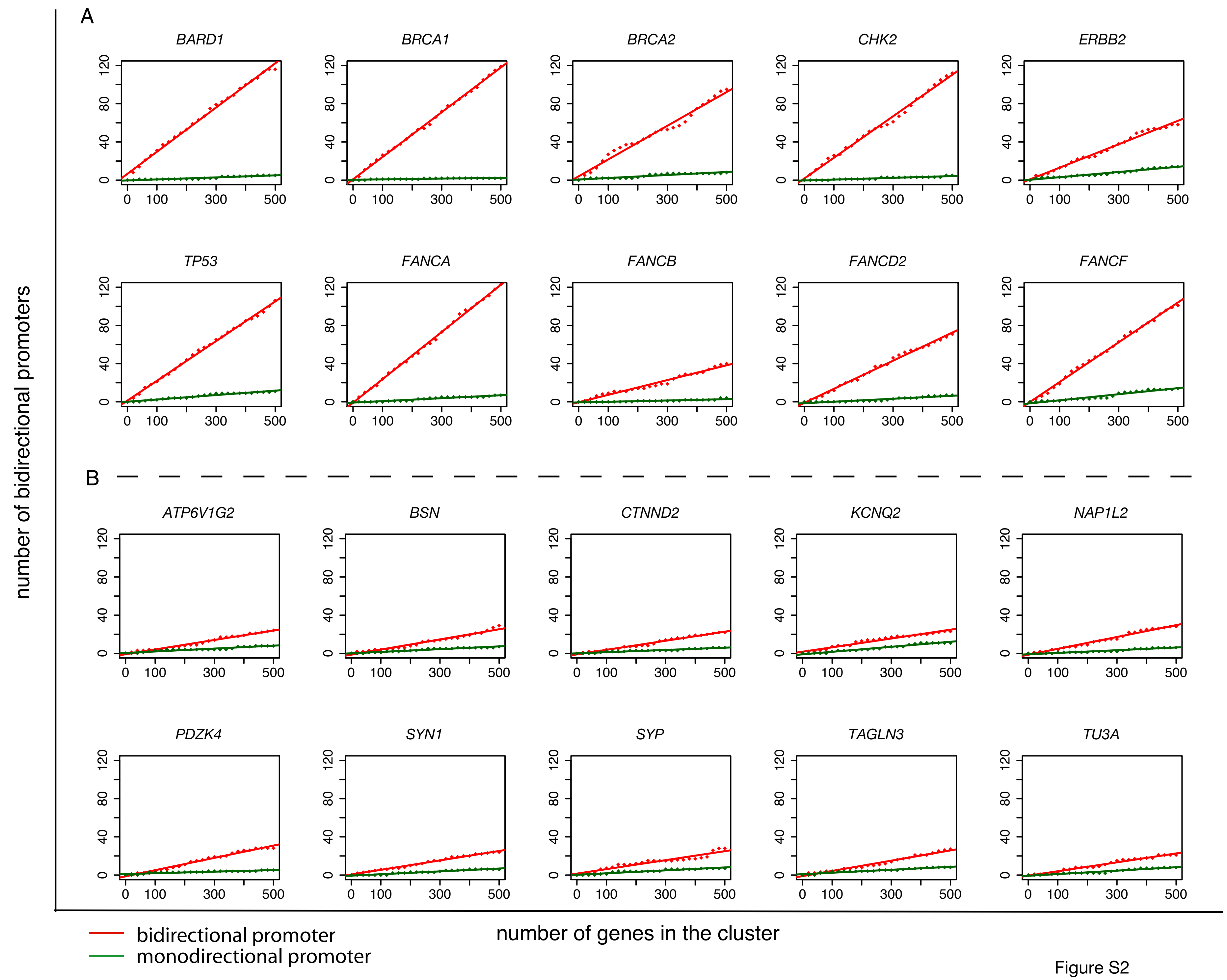

A
BARD1
BRCA1
BRCA2
CHK2
ERBB2
TP53
FANCA
FANCB
FANCD2
FANCF
B
ATP6V1G2
BSN
CTNND2
KCNQ2
NAP1L2
PDZK4
SYN1
SYP
TAGLN3
TU3A
number of bidirectional promoters
number of genes in the cluster
bidirectional promoter
monodirectional promoter

Figure S2
