# Supplementary material for: Comprehensive Annotation of Bidirectional Promoters Identifies Co-Regulation among Breast and Ovarian Cancer Genes
Source: PLoS Comput Biol. 2007 Apr 20;3(4):e72. doi: 10.1371/journal.pcbi.0030072 (PMC1853124; doi:10.1371/journal.pcbi.0030072)
Supplement: Supplementary file 3 [file pcbi.0030072.sg003.pdf]

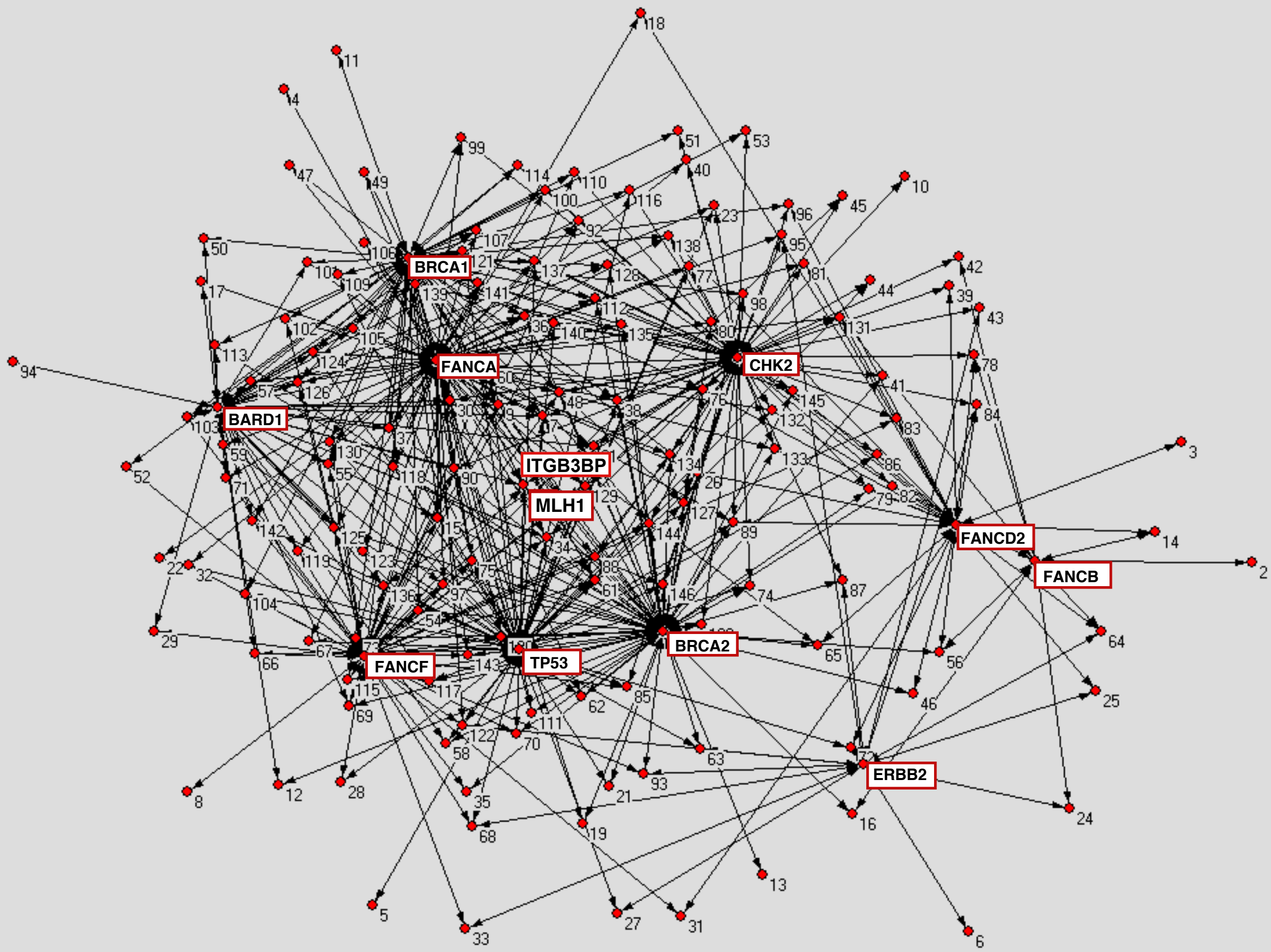
BRCA1
FANCA
CHK2
BARD1
ITGB3BP
MLH1
FANCD2
FANCB
BRCA2
FANCF
TP53
ERBB2

Figure S3
