# Supplementary material for: Comprehensive Annotation of Bidirectional Promoters Identifies Co-Regulation among Breast and Ovarian Cancer Genes
Source: PLoS Comput Biol. 2007 Apr 20;3(4):e72. doi: 10.1371/journal.pcbi.0030072 (PMC1853124; doi:10.1371/journal.pcbi.0030072)
Supplement: Supplementary file 4 [file pcbi.0030072.st001.doc]

Table S1. Endometrial Cancer genes with bidirectional promoters

| Bidirectional promoters | Lack bidirectional promoters |
| --- | --- |
| *P53* | *BRAF* |
| *ERBB2* | *KRAS* |
| *STK15* | *PTEN* |
| *MLH1* | *CDC4* |
| *PIK3CA* | *MSH2* |
| *PMS1* | *MSH6* |
| *PMS2* | *CTNNB1* |
| *CyclinE(2)* | *CDKN2A* |
