# Supplementary material for: Comprehensive Annotation of Bidirectional Promoters Identifies Co-Regulation among Breast and Ovarian Cancer Genes
Source: PLoS Comput Biol. 2007 Apr 20;3(4):e72. doi: 10.1371/journal.pcbi.0030072 (PMC1853124; doi:10.1371/journal.pcbi.0030072)
Supplement: Supplementary file 5 [file pcbi.0030072.st002.doc]

Table S2. Expression clusters over- and under-represented in bidirectional promoters.

| **Frequency of bidirectional promoters** | **Reference gene for the 500 member expression cluster** | **Over-represented category** | **p-value** |
| --- | --- | --- | --- |
| 0.56 | [*OVCA2*](http://bioinfo.weizmann.ac.il/cards-bin/carddisp?POLR2H) | mitochondrion | 1e-86 |
|  | [*DCXR*](http://bioinfo.weizmann.ac.il/cards-bin/carddisp?POLD2) | mitochondrion |  |
|  | *DGKD* | RNA processing |  |
|  | *AP2S1* | mitochondrion |  |
|  | [*PMPCB*](http://bioinfo.weizmann.ac.il/cards-bin/carddisp?POLR2J) | ribonucleoprotein |  |
| 0.55 | *DKC1* | RNA metabolism | 1e-100 |
|  | *RDBP* | RNA processing |  |
|  | *RNF4* | RNA processing |  |
|  | *SNRPD3* | RNA binding |  |
|  | *TPD52L2* | mitochondrion | 1e-52 |
|  | *UQCRC1* | mitochondrion | 1e-100 |
| 0.16 | *[OR2J3](http://gostat.wehi.edu.au/cgi-bin/goStatDisplay.pl?id=1145865512&displaytype=fullhtml&pcutoff=0.0001&ccutoff=30&mergecutoff=-1&method=both#OR2J3)* | Neurophysiological processing | 1e-59 |
| 0.17 | *CYP4B1* | Complement funciton | 1e-03 |
|  | *DARC* | Sensory perception | 1e-12 |
| 0.18 | *[PAQR7](http://gostat.wehi.edu.au/cgi-bin/goStatDisplay.pl?id=1145865512&displaytype=fullhtml&pcutoff=0.0001&ccutoff=30&mergecutoff=-1&method=both#CRYAB)* | Sensory perception | 1e-100 |
|  | *[ERBB3](http://gostat.wehi.edu.au/cgi-bin/goStatDisplay.pl?id=1145865512&displaytype=fullhtml&pcutoff=0.0001&ccutoff=30&mergecutoff=-1&method=both#ERBB3)* | Intrinsic to membrane | 1e-54 |

Note- Note- 16,087 reference genes were clustered into expression groups with their 500 most-similarly expressing genes. The frequency of bidirectional promoters was determined for each group as described in Methods. The groups were then sorted by the ratio of bidirectional promoters. Those with the highest and lowest ratios are shown in this table. The p-value was calculated using the GOSTAT server [http://gostat.wehi.edu.au](http://gostat.wehi.edu.au/). The highest frequency of bidirectional promoters was found for the gene functions of **RNA processing and mitochondrion,** which are statistically overrepresented in this selection of genes. The lowest frequencies were associated with **neurophysiological processing** and **sensory perception**. The entries for high and low ratios are separated by a bold line. A compete listing of all gene clusters can be provided by the authors upon request.
