# Supplementary material for: Comprehensive Annotation of Bidirectional Promoters Identifies Co-Regulation among Breast and Ovarian Cancer Genes
Source: PLoS Comput Biol. 2007 Apr 20;3(4):e72. doi: 10.1371/journal.pcbi.0030072 (PMC1853124; doi:10.1371/journal.pcbi.0030072)
Supplement: Supplementary file 6 [file pcbi.0030072.st003.doc]

Table S3. Bidirectional promoters in breast and ovarian cancer gene co-expression clusters.

| Breast or ovarian cancer gene | Ratio of bidirectional promoters in cluster |
| --- | --- |
| *BARD1* | 0.52 |
| *FANCA* | 0.48 |
| *FANCF* | 0.48 |
| *TP53* | 0.48 |
| *BRCA1* | 0.46 |
| *CHK2* | 0.46 |
| *BRCA2* | 0.41 |
| *FANCD2* | 0.32 |
| *ERBB2* | 0.29 |
| *FANCB* | 0.24 |
